# Supplementary material for: Wounds of Companion Animals as a Habitat of Antibiotic-Resistant Bacteria That Are Potentially Harmful to Humans—Phenotypic, Proteomic and Molecular Detection
Source: Int J Mol Sci. 2024 Mar 8;25(6):3121. doi: 10.3390/ijms25063121 (PMC10970316; doi:10.3390/ijms25063121)
Supplement: Supplementary file 1 [file ijms-25-03121-s001.zip › Supplementary Figure 1.pdf]

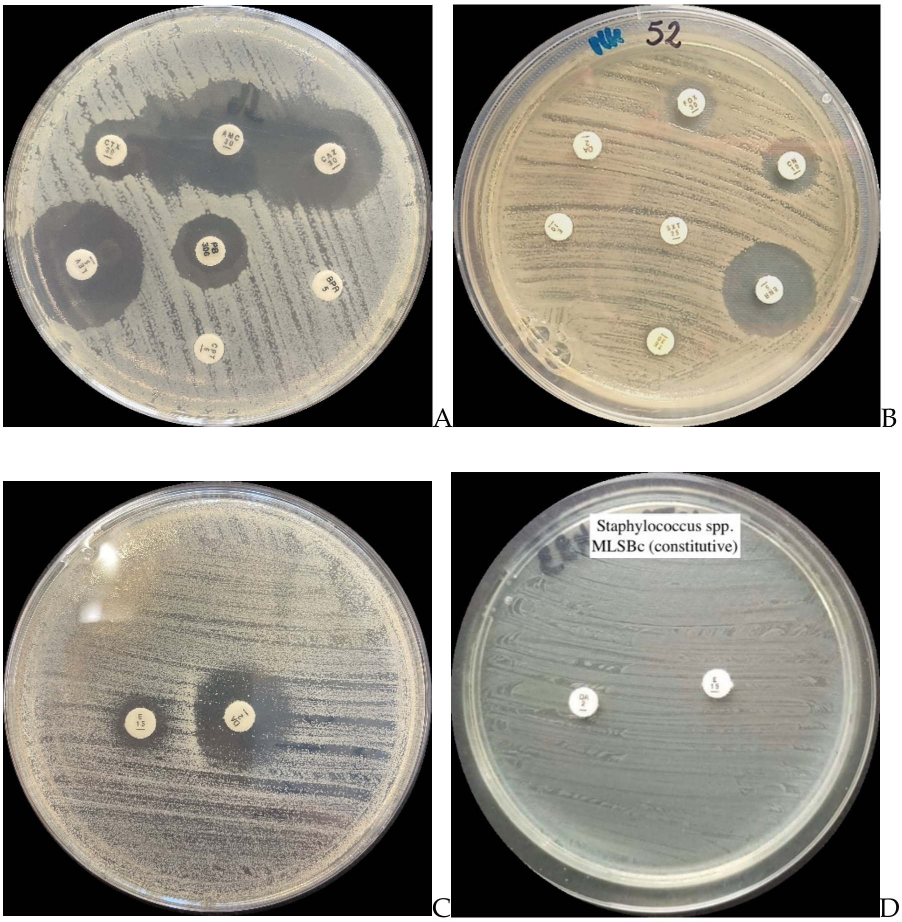

Figure S1. The resistance phenotypes observed in the examined groups of bacteria. A – extended spectrum beta lactamases (ESBL) in *Enterobacterales;* B – methicillin resistance in *Staphylococcus pasteuri*; C – inducible resistance to macrolides, lincosamids and streptogramins b (MLSb) in *Staphylococcus pseudintermedius*; D – constitutive MLSb in *Staphylococcus spp.*.
