# Supplementary material for: Wounds of Companion Animals as a Habitat of Antibiotic-Resistant Bacteria That Are Potentially Harmful to Humans—Phenotypic, Proteomic and Molecular Detection
Source: Int J Mol Sci. 2024 Mar 8;25(6):3121. doi: 10.3390/ijms25063121 (PMC10970316; doi:10.3390/ijms25063121)
Supplement: Supplementary file 1 [file ijms-25-03121-s001.zip › Supplementary Table 1.pdf]

Table S1. Species of bacteria isolated from wounds of three groups of animals.

Species	total (n=136)	cats (n=52)	dogs (n=80)	rabbits (n=4)
Gram-negative	65 (47.79)	23 (44.23)	38 (47.50)	4 (100)
Acinetobacter baumanni	1 (0.74)	1 (1.92)	0	0
Acinetobacter calcoaceticus	1 (0.74)	0	1 (1.25)	0
Acinetobacter johnsonii	1 (0.74)	1 (1.92)	0	0
Acinetobacter pittii	2 (1.47)	0	1 (1.25)	1 (25)
Acinetobacter ursingii	5 (3.68)	5 (9.62)	0	0
Aeromonas media	1 (0.74)	0	1 (1.25)	0
Brevundimonas diminuta	2 (1.47)	1 (1.92)	1 (1.25)	0
Citrobacter freundi	3 (2.21)	2 (3.85)	1 (1.25)	0
Enterobacter cloacae	1 (0.74)	1 (1.92)	0	0
Enterobacter hormaechei	2 (1.47)	0	2 (2.50)	0
Escherichia coli	11 (8.09)	1 (1.92)	8 (10.0)	2 (50)
Hafnia alvei	1 (0.74)	0	1 (1.25)	0
Klebsiella pneumoniae	2 (1.47)	0	1 (1.25)	1 (25)
Klebsiella spp.	1 (0.74)	1 (1.92)	0	0
Leclercia adecarboxylata	1 (0.74)	0	1 (1.25)	0
Pantoea agglomerans	2 (1.47)	0	2 (2.50)	0
Pantoea sp.	1 (0.74)	0	1 (1.25)	0
Moraxella osloensis	1 (0.74)	1 (1.92)	0	0
Proteus mirabilis	3 (2.21)	0	3 (3.75)	0
Proteus vulgaris	5 (3.68)	4 (7.69)	1 (1.25)	0
Pseudomonas aeruginosa	3 (2.21)	0	3 (3.75)	0
Pseudomonas putida	3 (2.21)	1 (1.92)	2 (2.50)	0
Pseudomonas graminis	1 (0.74)	0	1 (1.25)	0
Pseudomonas fulva	1 (0.74)	0	1 (1.25)	0
Pseudomonas koreensis	1 (0.74)	1 (1.92)	0	0
Psychrobacter sanguinis	2 (1.47)	0	2 (2.50)	0
Psychrobacter pulmonis	1 (0.74)	0	1 (1.25)	0
Serratia marcescens	3 (2.21)	3 (5.77)	0	0
Serratia liquefaciens	1 (0.74)	0	1 (1.25)	0
Stenotrophomonas maltophilia	2 (1.47)	0	2 (2.50)	0
Gram-positive	71 (52.21)	29 (55.77)	42 (52.50)	0
Bacillus pumilus	1 (0.74)	0	1 (1.25)	0
Curtobacterium flaccumfaciens	1 (0.74)	0	1 (1.25)	0
Enterococcus avium	1 (0.74)	0	1 (1.25)	0
Enterococcus faecalis	11 (8.09)	3 (5.77)	8 (10)	0
Enterococcus faecium	3 (2.21)	2 (3.85)	1 (1.25)	0
Enterococcus hirae	2 (1.47)	1 (1.92)	1 (1.25)	0
Kocuria rhizophila	1 (0.74)	1 (1.92)	0	0
Lactococcus garvieae	1 (0.74)	0	1 (1.25)	0
Lysinibacillus fusiformis	1 (0.74)	0	1 (1.25)	0
Macrococcus canis	1 (0.74)	0	1 (1.25)	0
Micrococcus luteus	1 (0.74)	0	1 (1.25)	0
Microbacterium maritypicum	1 (0.74)	0	1 (1.25)	0
Microbacterium oxydans	1 (0.74)	0	1 (1.25)	0

Microbacterium paraoxydans	1 (0.74)	1 (1.92)	0	0
Microbacterium phyllosphaerae	1 (0.74)	0	1 (1.25)	0
Peribacillus simplex	1 (0.74)	1 (1.92)	0	0
Staphylococcus aureus	5 (3.68)	4 (7.69)	1 (1.25)	0
Staphylococcus capitis	2 (1.47)	2 (3.85)	0	0
Staphylococcus cohnii	1 (0.74)	1 (1.92)	0	0
Staphylococcus devriesei	1 (0.74)	0	1 (1.25)	0
Staphylococcus condimenti	2 (1.47)	2 (3.85)	0	0
Staphylococcus epidermidis	2 (1.47)	1 (1.92)	1 (1.25)	0
Staphylococcus felis	2 (1.47)	2 (3.85)	0	0
Staphylococcus haemolyticus	1 (0.74)	0	1 (1.25)	0
Staphylococcus pasteuri	1 (0.74)	0	1 (1.25)	0
Staphylococcus lentus	1 (0.74)	1 (1.92)	0	0
Staphylococcus pseudintermedius	13 (9.56)	3 (5.77)	10 (12.5)	0
Staphylococcus schleiferi	2 (1.47)	0	2 (2.50)	0
Staphylococcus saprophyticus	1 (0.74)	0	1 (1.25)	0
Staphylococcus warneri	1 (0.74)	1 (1.92)	0	0
Staphylococcus sciuri	1 (0.74)	0	1 (1.25)	0
Streptococcus canis	5 (3.68)	2 (3.85)	3 (3.75)	0
