# Supplementary figures and images for: Wounds of Companion Animals as a Habitat of Antibiotic-Resistant Bacteria That Are Potentially Harmful to Humans—Phenotypic, Proteomic and Molecular Detection
Source: Int J Mol Sci. 2024 Mar 8;25(6):3121. doi: 10.3390/ijms25063121 (PMC10970316; doi:10.3390/ijms25063121)

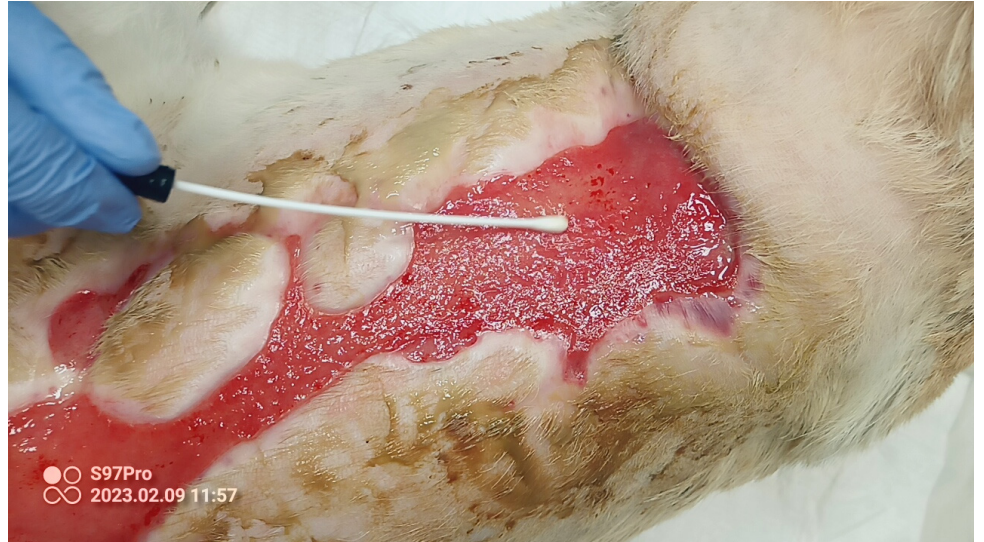

**Figure S2.** Collection of a swab sample from a skin wound of a dog.

Supplement: Supplementary file 1 [file ijms-25-03121-s001.zip › Supplementary Figure 2.pdf]
